# Knowledge, Attitude, and Associated Factors towards Colostrum Feeding among Antenatal Care Attendant Mothers in Gununo Health Centre, Wolaita Zone, Ethiopia 2019: Cross-Sectional Study

**DOI:** 10.1155/2020/3453502

**Published:** 2020-01-21

**Authors:** Addisu Yeshambel Wassie, Natnael Atnafu Gebeyehu, Kelemu Abebe Gelaw

**Affiliations:** Department of Midwifery, College of Health Science and Medicine, Wolaita Sodo University, Wolaita Sodo, Ethiopia

## Abstract

**Background:**

The role of colostrum in promoting the growth and development of the newborn as well as fighting infections is widely acknowledged. In Ethiopia, there are differences in cultures in the acceptability of colostrum and the prevalence of colostrum feeding. Although breastfeeding is a common practice in Ethiopia, there is a difference in the awareness and attitude of pregnant mothers regarding colostrum feeding.

**Objectives:**

To assess knowledge, attitude, and associated factors towards colostrum feeding among antenatal care attendant mothers in Gununo Health Center, South Ethiopia, 2019.

**Methods:**

Facility-based cross-sectional study design was conducted among 342 ANC (antenatal care) attendant mothers in Gununo Health Center from April to May 2019. Data was collected by using structured interviewer questionnaires and the subjects were selected through systematic random sampling. Data template was prepared by Epi data-manger version 4.2 and SPSS version 23 was used for analysis. Bivariate and multivariate analysis with 95% CI was employed. Variables found to have a *p*-value < 0.2 in the binary logistic regression were entered into multivariate analysis and strength of association was declared at *p*-value < 0.2 in the binary logistic regression were entered into multivariate analysis and strength of association was declared at

**Results:**

Among the study participants 226 (66.1%) were knowledgeable and 39 (11.4%) were not knowledgeable on colostrum feeding. From the respondents, 239 (69.9%) had a positive attitude and the rest 103 (30.1%) mothers had a negative attitude towards colostrum feeding. Respondents who had more than four children (AOR = 1.21, 95% CI [1.31, 2.47], ANC visit (four times and above) (AOR=2.8, 95% CI [2.23, 4.49]), and counseled about colostrum feeding (AOR = 2.29, 95% CI [2.34, 3.74]), were some variables that significantly associated with knowledge of colostrum feeding. Those who had been counseled about breastfeeding (AOR = 1.16, 95% CI [1.59–3.96]), ANC visit (AOR = 11.32, 95% CI [1.14, 112.64]), and multiparas (AOR = 5.68, 95% CI [1.57, 20.53]) were some variables that significantly associated with attitude. *Conclusion and Recommendation.* Even though the mothers' knowledge and attitude seem higher than from previously conducted articles in Ethiopia, still gaps were seen clearly on colostrum feeding in the area. It is recommended to set strategies to promote colostrum feeding.

## 1. Introduction

Colostrum is the first milk or a sticky white or yellow fluid secreted by the breast during the second half of the pregnancy and for a few days (3-4) after birth before the regular breast milk comes. It is a concentrated form of ‘immature milk', which is very high in protein, antibodies, and other protective components that are important for your newborn [1]. The first milk is the most suitable food for the newborn, universally acknowledged as the perfect 1^st^ food for infants and a suggested regimen for expressing and storing of colostrum during pregnancy is included with advice about skin-to-skin contact in the first 24 hours to maximize breast milk output in the long term [2]. Many articles reveal that bacterial, viral, fungal, and protozoal infection of the newborn baby can be reduced by feeding colostrum and advantages to the mother's health by increasing the postpartum infertility period, helping them return to their pre-gestational weight, and reducing their risk of breast and ovarian cancer [3].

Colostrum feeding is associated with a reduced risk of otitis media, gastroenteritis, and respiratory illness, necrotizing entero-colitis, obesity, and hypertension [4]. In the developing countries where the rate of communicable diseases is high, timely provision of colostrum is reducing diarrheal disease in the neonates [5]. The study conducted in India on timely initiation of breastfeeding is recognized as the first and vital step toward reducing mortality in infants and children under-five years of age. It has the potential to prevent 16−22% of neonatal deaths with immediate breastfeeding after birth [6]. Early breastfeeding initiation is practiced by 39.6%, 83.7%, 47.3%, and 62.9% of women in Amibara district [7], Dale woreda [8], Gurage zone [9], and Debre Birhan [10] respectively.

Globally, more than 4000 infants and young children die because they do not get colostrum within the first hour after birth. Most of the infants are given liquids other than their mother's milk in the first few days after birth. The rate of breastfeeding varies in communities from almost 70% to a low of 13% as this is culturally influenced [11]. Among women in developing countries who do not give colostrum feeds, most of them avoid colostrum feeding based on traditional or cultural beliefs that range from no nutritional value for infants to harmful for the infant's health. Some women may specify no reason for avoiding colostrum rather than tradition [12].

Ethiopia has one of the highest infant mortality rates in the world that occur due to inappropriate neonatal feeding. Although breastfeeding is almost universal across all Ethiopian ethnic groups and geographical areas, it does not always meet the WHO/UNICEF recommendations [13]. Avoiding colostrum in the first 3-4 days increases the risk of infection and death among neonates [14]. Colostrum avoiding decreases the new-borns' nutrients and immunoglobulin, causing a reduction in the priming of the gastrointestinal tract, and also increases the risk of infant morbidity and mortality [15]. Although colostrum feeding provides new-borns with immunity to infection, any practice that reduces a frequency or volume of breastfeeding during this time could reduce the neonates' long-term health and immunological defense [16]. Even though the world health organization (WHO) recommended to initiate colostrum feeding within the first hour after birth, a higher number of mothers avoided their colostrum before giving milk to their neonates [17]. According to different studies, children who did not receive colostrum are more likely to develop many infections, stunting, underweight, and wasting [18–20].

Many studies have identified several factors that influence the colostrum feeding in developing countries, although there are few studies regarding the factors affecting the timely initiation of colostrum feeding. These factors include, residence, maternal education, age, occupation, religion, marital status, income, and having obstetric history like parity, number of child alive, heading household, support from family, previous delivery, baby illness, influence from others, number of ANC (antenatal care), visit history of previous delivery and mode of delivery, and counseled about breastfeeding [7, 9, 10, 21, 22] were found to be predictors that either positively or negatively influence timing, knowledge, and attitude of colostrum feeding.

Even if there is a steady decline in Neonatal and infant morbidity and mortality in Ethiopia, it is still a major health problem. Studies were done in West Gojjam and Gondar indicated that neonatal mortality was as high as 18.6 and 48.3 per 1000 live births respectively and colostrum feeding is still unsatisfactory/low [23, 24] and infant mortality was still 48 deaths per 1,000 live births in the country [25]. In Ethiopia, the major neonatal deaths that occurred in the first week of life can be reduced by immediate initiation of breastfeeding and colostrum after birth [2, 18, 26]. Therefore, this study was aimed to assess the knowledge and attitude of colostrum feeding and associated factors among ANC attendant mothers in Gununo Health Centre.

## 2. Methods

### 2.1. Study Area and Setting

Gununo town is found in the Southern, Nations, Nationalities, and People's Region (SNNPR), it is the capital town of Damot Sore Woreda Wolaita Zone. It is 345 kms far from Addis Ababa which is the capital city of Ethiopia, and 17 kms away from the capital Zone Wolaita Sodo. Located on the South by Sodo Zuriya, on the North West by Boloso Bombe, on the West by Kindo Koysha, and on the East by Boloso Sore. Based on the 2011 E.C Population profile of Gununo Health Center, this town has a total population of 27,297. From this 13,376 are men and 13,921 are women. The reproductive age group of women in this town are about 23.3% (6360). There is only one health center and three private clinics in the town [27].

### 2.2. Study Design and Period

A facility-based cross-sectional study was conducted among ANC attendant mothers from January to May 2019 in Gununo Health Center, Gununo town, Ethiopia.

#### 2.2.1. Source Population

All pregnant mothers who attend ANC in Gununo Health Center.

#### 2.2.2. Study Population

All selected pregnant mothers attending ANC in Gununo Health Center at the time of data collection.

#### 2.2.3. Inclusion Criteria

All pregnant mothers coming for ANC service during data collection were included.

#### 2.2.4. Exclusion Criteria

Those pregnant mothers who were involuntary and severely ill during data collection period were excluded.

### 2.3. Sample Size Determination

The sample size was determined using a single population proportion formula by considering proportion (*P*) on colostrum feeding from the previous study conducted in MizanTepi of SNNPR showed that 65.2% for knowledge and 69.4% for attitude [28].

*n* = sample size.

*Z* = level of confidence (1.96)2 CI-95%.

*P* = proportion of pregnant mothers on colostrum feeding from the previous study conducted in Mizan Tepi of SNNPR.

*d* = margin of error (5%).

Calculation:

For knowledge = **65.2%**

(1) $$\begin{array}{c} ni=\frac{{\left(z\alpha /2\right)}^{2}p\left(1-p\right)}{d^{2}}=\frac{{\left(1.96\right)}^{2}0.652\left(1-0.652\right)}{0.05^{2}}=348. \end{array}$$

Since the source population was less than 10,000 which is 6360, we used the reduction formula as follows:

(2) $$\begin{array}{c} nf=\frac{ni}{1}+\frac{ni}{N}, \end{array}$$

where,

*N* = Source population- estimated as reproductive age group of women,

*nf* = required sample size,

*ni* = calculated sample size.

Hence, the sample size was calculated at a total of source population *N* = 6360 and *ni* = 348, and *nf* = 330. With 5% of the nonresponse rate, the total sample size was **346 **pregnant mothers.

### 2.4. Sampling Technique

A systematic random sampling technique was conducted to select the study subjects. The first patient flow in the preceding six months was revised from the ANC registration book and the average client flow was 471 mothers. The result was divided by the sample size and the k^th^ value was obtained. Then from the k^th^ value, we selected one mother from the 2 (two) by the lottery method. This might be taken as the first respondent in our study and the data was collected every k^th^interval (*n* = 471/346 = 2).

#### 2.4.1. Outcome (Dependent) Variables

  (i) Knowledge of colostrum feeding.
  (ii) Attitude towards colostrum feeding.

#### 2.4.2. Exposure (Independent) Variables

 
*(i) Maternal socio-demographic and economic variables* (Age, marital status, residence, occupation, maternal educational status, religion, ethnicity, and monthly income).
 
*(ii) Maternal Obstetric and Health Services related variables* (Para, number of child alive, ANC care and number of ANC visit, Counseling on breastfeeding at ANC, history of home delivery and delivery attendant, history of previous delivery and mode of delivery, heading household, history of influence of somebody on breastfeeding and who influenced it, history support from family on breastfeeding, history of baby illness with 4 days of delivery, and history of birth and type of delivery).

#### 2.4.3. Operational Definition

*Colostrum*: The breast milk produced in the first 3-4 days after birth.

*Knowledgeable*: The answers greater than 75% of the questions out of the total knowledge related questions.

*Fairly knowledgeable: *The answers between 25−75% of the questions out of the total knowledge related questions.

*Not knowledgeable: *The answers less than 25% of the questions out of the total knowledge related questions.

*Positive attitude*: Those who answered positively greater than 60% of the attitude related questions.

*Negative attitude*: Those who answered negatively less than 60% of the attitude related questions [21, 28].

#### 2.4.4. Data Collection Instrument

Semi-structured questionnaires amended from the USAID tool was used. First, it was translated to Amharic by an expert of the language and then it was translated back to English for checking correctness.

#### 2.4.5. Data Collection Method

Data were collected by five BSc Midwife's and it was supervised by the principal investigator. During data collection, first consent was asked. The first mother was selected using the lottery method. An exit interview was conducted in a separate room that keeps the privacy and confidentiality of the information. The collected data was secured to keep their privacy and confidentiality.

#### 2.4.6. Data Quality Control

To assure the quality of data, pretest of the instrument was done before the actual data collection among 5% (18) of the study subjects in the health center other than the study area which was similar to the study population and the necessary modifications and correction was made to standardize and ensure its validity. Completeness of data was checked every day after the data collection was completed.

#### 2.4.7. Data Processing and Analysis

First, the collected data were checked manually for completion and any incomplete or misfiled questions. Then the data was cleaned and stored for consistency and entered into the EpiData version 4.2, and then it was exported to the Statistical Package for the Social Sciences (SPSS) version 23.0 software for analysis.

Descriptive statistics like frequency, proportion, mean, and standard deviation were computed to describe study variables in relation to the population. Logistic regression (bivariate and multivariate) was used to determine the effect of independent variables on the outcome variables. The strength of the association was declared at *p*-value<0.05. The variables found to have a *p*-value<0.2 in the binary logistic regression were entered/exported into the multivariate analysis to identify their independent effects and the final results were presented as odds ratio (OR). Finally, the results were compiled and presented using texts, tables, and graphs.

#### 2.4.8. Ethical Considerations

Ethical clearance was obtained from the ethical review committee of the Wolaita Sodo University. A formal letter, from the research and review committee of the faculty of medicine and health sciences, was submitted to the Gununo Health Center and concerned bodies to obtain their cooperation.

The purpose of the study was explained to the study subjects at the time of data collection and verbal consent was taken from the participants to confirm whether they were willing to participate. The confidentiality of the respondents was ensured throughout the research process.

## 3. Result

### 3.1. Socio-Demographic Characteristics of the Respondents

A total of 342 mothers participated in this study with a response rate of 98.8%. Among the mothers interviewed, 133 (38.9%) mothers were between the age group of 25−29 followed by 91 (26.6%) mothers who were between 20−24. More than half of the mothers, 177 (51.8%) were living in a rural area. Among the respondents, 321 (93.9%) were married followed by 11 (3.2%) who were divorced. Most of the participants, 286 (85.1%) were Wolaita ethnically followed by Hadiya 34 (10.4%). The majority of the respondents 231 (67.8%) were Protestants followed by Orthodox 89 (26%). Around, 114 (33.8%) had complete primary school. Most mothers 144 (43%) were self-employed followed by 111 (33.1%) who were housewives. More than half of the participants, 213 (62.3%) had a monthly income of less than 500 ETB (Table 1).

### 3.2. Maternal Experience

Out of the total participants, 197 (57.6%) of them were multipara and 154 (46.2%) had around 1-2 live child/children (Table 2).

Nearly three fourth, 283 (83%) of the participants reported that as they had given/fed the fluid that came from the breast within 3 days of delivery for their baby followed by water, sugar (34 (10%)), butter 22 (6.5%), and the remaining participants 3 (0.9%) had gave nothing to their babies.

### 3.3. Knowledge of Respondents on Colostrum Feeding

According to the set criteria regarding knowledge of respondents toward colostrum feeding, 226 (66.1%) were knowledgeable followed by fairly knowledgeable which was 77 (22.5%), and the remaining 39 (11.4%) were poorly knowledgeable.

From the total respondents, majority of the mothers, 180 (59.8%) believed that colostrum is important for the baby to prevent illness, followed by 101 (33.7%) were reported as it will be important for the growth of the baby and the remaining 20 (6.6%) were reported as it will have the tendency to catch a cold. The majority, 283 (86.3%) believe that starting breastfeeding one hour after delivery is important, 34 (10.4) initiated breastfeeding their child within six hours after delivery (Table 3).

### 3.4. Results on the Attitude of the Respondents Towards Colostrum Feeding

According to a set criteria regarding the attitude of the respondents towards colostrum feeding the majority of mothers, 239 (69.9%) had a positive attitude toward colostrum and the remaining 103 (30.1%) had a negative attitude (Table 4).

### 3.5. Socio-Economic Variables, Obstetric Service-Related Factors Associated with Knowledge Colostrum Feeding (Table 5)

To assess the association of each independent variable with knowledge of colostrum feeding the Binary Logistic regression was performed. The factors that showed a *p*-value of less than 0.2 were added to the multivariate regression model. The result revealed that on the bivariate analysis: residence, number of living children, number of ANC visit, history of previous assisted delivery, counseled about breastfeeding during ANC follow-up, heading household/s, faced influence from somebody about breastfeeding, prior-birth, history of support from their family about breastfeeding, history of baby illness in the first 4 days of delivery, and time of initiation of breastfeeding was significantly associated with knowledge of colostrum feeding. In multivariate logistic regression, number of children alive, number of ANC visit, counseled about breastfeeding during ANC follow-up, history of support from their family about breastfeeding, and history of baby illness in the first 4 days of delivery were significantly associated with the knowledge of colostrum feeding at *p*-value of <0.05 (Table 5). Respondents who had greater than four children were 1.21 times more likely knowledgeable on colostrum feeding than with one to two children (AOR = 1.21, 95% CI [1.31−2.47]). From the participants, those who visit ANC four times and above were 2.8 times more likely knowledgeable than those who visit less or equal to one ANC visit (AOR = 2.8, 95% CI [2.23−4.49]). Similarly, those respondents who were counseled about colostrum feeding during ANC follow-up were 2.29 times knowledgeable on colostrum feeding (AOR = 2.29, 95% CI [2.34–3.74]. In addition, those who got support from their family about colostrum feeding were 12 times more likely knowledgeable than those who did not get support (AOR=12, 95% CI [1.24–5.78]) whereas, those mothers having baby illness within the first 4 days of delivery were 33% less likely to give colostrum to their baby's than others (AOR = 0.67, 95% CI [0.01, 0.94]) (Table 5).

### 3.6. Socio-Economic Variables, Obstetric Service-Related Factors Associated with Attitude of Colostrum Feeding (Table 6)

To assess the association of each independent variable with the attitude of colostrum feeding, Binary Logistic regression was performed. The factors that showed a *p*-value of less than 0.2 were added to the multivariate regression model. The result revealed that on bivariate analysis: age of respondents, residence, educational status, religion, parity, number of children alive, number of ANC visit, counseled about breastfeeding during ANC follow-up, prior-birth, history of support from their family about breastfeeding, and history of baby illness in the first 4 days of delivery were significantly associated with the attitude of colostrum feeding. In multivariate logistic regression parity, the number of ANC visit counseled about breastfeeding during ANC follow-up, Prior-birth, and history of support from their family about breastfeeding were significantly associated with the attitude of colostrum feeding at *p*-value of <0.05 (Table 6). Respondents who were counseled about breastfeeding were 1.16 times more likely to practice a positive attitude towards colostrum feeding compared with not counseled (AOR = 1.16, 95% CI [2.59–3.96]). From the participants, those who visit ANC three times and above were 11.32 times more likely have positive attitude towards colostrum feeding than from those who visit less or equal to one ANC visit (AOR = 11.32, 95% CI [1.14, 112.64]) and multiparas were positive attitude than primiparas (AOR = 5.68, 95% CI [1.57, 20.53]). Similarly, those respondents who had prior-birth were more likely to have a positive attitude than others (AOR = 4.72, 95% CI [1.69, 13.21]). However, those mothers who got support from their family about colostrum feeding had a negative attitude towards colostrum feeding (AOR = 0.22, 95% CI [0.11–0.43]) (Table 6).

## 4. Discussion

This study investigated the knowledge, attitude, and associated factors towards colostrum feeding among pregnant mothers attending ANC in Gununo health center, Wolaita Zone, Ethiopia. In this study, 66.1% of mothers were knowledgeable, 22.1% were fairly knowledgeable however the remaining (11.4%) of mothers were not knowledgeable. The study was lower than the study conducted in Kolhapur, Dhaka city which showed that 77% of mothers had knowledge about colostrum feeding (27) and the study done in Nepal which showed 74% knowledgeable (30). The difference might be due to the difference in the socio-economic background of the participants and the sample size used. And this study was in line with the study conducted in Mizan Tepi, Bench Maji Zone, SNNP Region which showed that 65.2% had knowledge about colostrum feeding (29). The similarity might be due to socio-cultural similarities or/and awareness that was created on the advantages of colostrum feeding by health care providers in the country.

The study conducted in Nepal showed that 69% of the women participants knew colostrum as nutritious milk to be fed to the new babies, 41% had knowledge that it helps in proper growth of babies and fight against infection, 9% reported as proper growth (30) which is comparable with this study which showed that 59.8% were reported colostrum as to prevent illness, 33.7% as it will be important for growth and the remaining 6.6% reported it as it has high tendency to protect babies from cold. This might be due to increased information dissemination and awareness creation done for mothers on the advantages of colostrum feeding by the health care providers.

According to this study finding, 86.3% of the respondents initiated colostrum feeding within 1 hour of delivery, 10.4% of the initiate within 6 hours. This is higher than the study done in the Amibara district, northwestern Ethiopia (19) which showed that only 39.6% of mothers initiated breastfeeding within 1 hour after delivery and the study conducted at Raya Kobo, Northern eastern Ethiopia 2014 (21), reported that colostrum is believed as the dirty part of milk by 25.9% of the respondents. The differences may be due to socio-cultural differences among the study area. The difference might be due to study time and study design differences.

The finding of this study indicated that among study participants 69.9% had positive attitude and the remaining 30.1% had negative attitude towards colostrum feeding, which is in line with the study conducted in Mizan Tepi of SNNPR (29) which showed 69.4% positive attitude towards colostrum feeding and lower than the study conducted in Nepal (30) which showed 89% were positive attitude towards colostrum feeding. This might be due to socio-economic differences and a better understanding of colostrum feeding among the study participants living in Nepal.

In this study finding, respondents who had greater than four children were 1.21 times more likely knowledgeable on colostrum feeding than with one to two children. From the participants, those who visit ANC four times and above were 2.8 times more likely knowledgeable than those who visit less or equal to one ANC visit. Similarly, those respondents who were counseled about colostrum feeding during ANC follow-up were 2.29 times knowledgeable on colostrum feeding (AOR = 2.29, 95% CI [2.34, 3.74]) .In addition, those who got support from their family about colostrum feeding were 12 times more likely knowledgeable than who did not get support (AOR = 12, 95% CI [1.24, 5.78]) whereas, those mothers having baby illness within 4 days of delivery were 33% less likely to give colostrum to their baby's than others (AOR = 0.67, 95% CI [0.01, 0.94]). The finding is comparable with the study done in Debre Berhan town, Ethiopia(10), showed not being counseled about timely initiation of breastfeeding during antenatal care (AOR 0.40; 95% CI 0.18, 0.88), a systematic review done in Ethiopia (30), showed antenatal care (Odds Ratio (OR) 0.25, 95% CI 0.09, 0.69), counselling on infant feeding (OR 0.37, 95% CI 0.22, 0.63), and timely initiation of breastfeeding (OR 0.28, 95% CI 0.21, 0.38), were significantly associated.

Similarly, in this study finding, respondents who was counseled about breastfeeding were 1.16 times more likely to practice a positive attitude towards colostrum feeding compared with not counseled (AOR=1.16, 95% CI [2.59–3.96]). From the participants, those who visit ANC three times and above were 11.32 times more likely have positive attitude towards colostrum feeding than from those who visit less or equal to one ANC visit (AOR = 11.32, 95% CI [1.14, 112.64]) and multiparas were positive attitude than primiparas (AOR = 5.68, 95% CI [1.57, 20.53]). Similarly, those respondents who had a history of previous delivery were more likely to have a positive attitude than others (AOR =  4.72, 95% CI [1.69, 13.21]). However, those mothers who got support from their family about colostrum feeding had a negative attitude towards colostrum feeding (AOR = 0.22, 95% CI [0.11–0.43]). The study was comparable with the study done in Arba Minch Zuria, Southern Ethiopia (22), which showed Ethiopia personnel support women at delivery time (AOR 0.52; 95% CI 0.21, 0.58) were inversely associated with delayed initiation of breastfeeding practices.

### 4.1. Strength of the Study

Since it was primary data, both Governmental and Nongovernmental organizations involved in the reduction of neonatal and infant morbidity and mortality can utilize the findings of this study as a means of the intervention of programs and for further study.

### 4.2. Limitation of the Study

The respondent may answer questions in a manner that would be viewed favorably by others (may under /inaccurately report their view towards colostrum feeding). Recall bias and courteous bias might not be minimized. Since the data is collected at a single point in time, a temporal relationship could not be established.

### 4.3. Conclusion and Recommendation

Finding from this study indicate that the mothers' knowledge and attitude seem high. However, further awareness is necessary to improve the knowledge and attitude towards colostrum feeding in Gununo health center for those with a negative attitude and not knowledgeable. Also, this study indicates that early initiation of complementary feeding before six months is high. Number of the child alive, parity, number of ANC visits, counseled about breastfeeding during ANC follow-up, history of support from their family about breastfeeding, and history of baby illness in the first 4 days of delivery were significantly associated with knowledge and attitude of colostrum feeding. The Health extension workers and Governmental and Nongovernmental organizations should give health education about the importance of colostrum feeding for the baby and its effect on the mothers as well as encourage mothers to attend antenatal care during pregnancy.

## Figures and Tables

**Table 1:** Socio-demographic characteristics of pregnant mothers attended ANC follow-up in the Gununo health center, SNNP region, Southern Ethiopia, 2019.

Variables	Category	Frequency	Percentage (%)
Age	15–19	35	10.2
	20–24	91	26.6
	25–29	133	38.9
	30–34	69	20.2
	>35	14	4.1

Residence	Urban	165	48.2
	Rural	177	51.8

Marital status	Single	11	3.2
	Married	321	93.9
	Divorced	7	2
	Widowed	3	0.9

Educational level	Cannot read and write	97	28.4
	Primary school	114	33.3
	Secondary school	61	17.8
	Diploma and above	70	20.5

Occupation	Self-employed	144	43
	Housewife	111	33.1
	Government employed	63	18.8
	Farmer	17	5.1
	Others(student)	7	2

Ethnicity	Wolaita	286	85.1
	Hadiya	34	10.4
	Kambata	16	4.8
	Others (slita, guragia and dawero)	6	1.8

Religion	Protestant	231	67.5
	Orthodox	89	26
	Muslim	17	5
	Catholic	5	1.5

Income	<500	213	62.3
	500–999	65	19
	1000–1499	44	12.9
	1500–1999	9	2.6
	2000–2500	9	2.6
	>2500	2	0.6

**Table 2:** Mothers distribution by the number of children they have at the Gununo health center, Wolaita zone, SNNP region, Southern Ethiopia, 2019.

Variables	Frequency	Percentage (%)
*Parity:*		
Primipara	145	42.4
Multipara	197	57.6

*Number of living children:*		
1-2	154	46.2
3-4	138	41.4
5 or more	41	12

**Table 3:** Maternal knowledge regarding colostrum feeding in Gununo health center, Wolaita zone, SNNP region, Southern Ethiopia, 2019.

Variable	Frequency	Percentage
*Colostrum:*		
Bad fluid from the breast in the first 4 days	17	5
Important breast milk in the first 4 days	291	85.1
I do not know	34	9.9

*Days colostrum will stay:*		
For 1 day	15	4.4
For 2 days	33	9.6
For 3 days	162	47.4
More than 3 days	132	38.6

*Does colostrum has an advantage to the babies:*		
Yes	297	86.6
No	16	4.7
I do not know	29	8.5

*Time of initiation of the 1^st^ milk:*		
Within one hour after delivery	283	86.3
Within six hours after delivery	34	10.4
After 24 hr after delivery	11	3.4
Others (I do not know)	14	4.4

*Additional food or drink should be given from birth to six months in addition to colostrum:*		
Nothing	147	43
Water	46	13.5
Cow's milk	149	43.9

**Table 4:** Mother's attitude towards colostrum feeding in Gununo Health Center, Wolaita Zone, SNNP Region, Southern Ethiopia, 2019.

Attitude towards colostrum feeding	Response	Frequency	Percentage
Colostrum feeding is important for the baby because it is the best food for growth	Agree	269	78.7
	I do not agree	25	7.3
	Neutral	5	1.5
	I do not know	43	12.6
Colostrum feeding is not good because it causes abdominal cramp and diarrhea	Agree	29	8.5
	I do not agree	240	70.2
	I do not know	58	17
	Neutral	15	4.4
Colostrum is not good for the child and forbidden by culture	Agree	7	2
	I do not agree	295	85.3
	I do not know	38	11
	Neutral	6	1.7
Colostrum is not important for the baby because of its dirty part of milk	Agree	21	6.1
	I do not agree	257	70.1
	Neutral	7	2
	I do not know	57	16.7

**Table 5:** Bivariate and multivariate logistic regression analysis of knowledge of colostrum feeding and its explanatory variables (*n* = 342) (N.B: yes = knowledgeable and no = not knowledgeable )).

Variables	Yes	No	OR (95% CI)	AOR (95% CI)
*Residence:*				
Urban	151	14	0.52 (0.28–0.96)^∗∗^	0.57 (0.29–1.12)
Rural	152	25	1.02 (0.53–2.00)	1.03 (0.50–2.13)

*Number of child alive:*				
1-2	135	19	1	1
3-4	127	11	0.36 (0.08–1.63)^∗^	0.6 (0.002–0.47)
>4	39	2	0.56 (0.13–2.90)	1.21 (1.31–2.47)^∗∗^

*Previous assisted delivery:*				
Health professional/s	6	2	3.2 (0.59–0.69)^∗^	2.34 (1.05–5.25)
Traditional birth attendant	126	13	1	6.08 (0.24–15.33)

*The number of ANC visit:*				
Once	59	4	1	1
Twice	85	15	29.5 (4.09–212.9)^∗∗∗^	1.17 (0.00–1.76)
Three times	113	9	11.3 (1.90–67.5)^∗∗∗^	7.73 (0.00–09.39)
Four times	44	7	25.1 (4.04–156.2)^∗∗∗^	2.80 (2.23–4.49)^∗∗^
I have no follow-up	2	4	12.57 (1.93–82.0)^∗∗∗^	3.12 (0.03–1.09)

*Counseled about breast feeding during ANC follow-up:*				
Yes	211	9	0.13 (0.6–0.29)^∗∗∗^	2.29 (2.34–3.74)^∗∗^
No	92	30	1	1

*Heading household/s:*				
Yes	64	239	0.31 (1.00–1.04)^∗∗^	0.65 (0.34–12.6)
No	3	36	1	1

*Prior-birth:*				
Yes	287	23	0.8 (0.36–0.18)^∗∗∗^	16.2 (0.00–2.12)
No	16	16	1	1

*History of support from their family about breastfeeding:*				
Yes	204	2	0.26 (0.01–0.11)^∗∗∗^	12 (1.24–5.78)^∗∗^
No	99	37	1	1

*History of baby illness in the 1^st^ 4 days of delivery:*				
Yes	71	2	0.18 (0.42–0.75)^∗∗^	0.67 (0.01–0.94) ∗∗
No	232	37	1	1

*Time of initiation of breastfeeding:*				
Within 1 hr	248	14	1	1
After 2 hrs	46	10	50.6 (5.85–438.02)^∗∗∗^	4.16 (0.29–24.9)
After 3 hrs	6	0	1.78 (0.00)	2.04 (0.9–35.5)
After 4 hrs	4	2	5.5 (0.39–78.6)	3.04 (1.09–27.09)

NB: ^∗ ^= *p*-value <0.2, ^∗∗ ^= *p*-value <0.05, ^∗∗∗^ = *p*-value <0.01

**Table 6:** Bivariate and multivariate logistic regression analysis of attitude colostrum feeding and its explanatory variables (*n* = 342).

Variables	Yes	No	OR (95% CI)	AOR (95% CI)
*Age:*				
<20	18	17	1	1
20–24	61	30	1.06 (0.56–2.05)	0.17 (0.24–1.23)
25–29	94	39	2.03 (1.31–3.15)^∗∗∗^	0.34 (0.66–1.94)
30–34	53	16	2.41 (1.66–3.50)^∗∗∗^	0.48 (0.99–2.36)
>34	13	1	3.31 (1.89–5.79)^∗∗∗^	1.26(0.26–6.06)

*Residence:*				
Urban	118	47	1.64 (1.41–1.89) ∗∗∗	1.07 (0.55–2.07)
Rural	121	56	1	1

*Educational status:*				
Cannot read a& write	65	32	1	1
Completed 1–8	77	37	2.03 (1.33–3.10)^∗∗∗^	1.34(0.51–3.54)
Completed 9–12	43	18	2.08 (1.41–3.08)^∗∗∗^	1.79 (0..75–4.31)
Diploma and above	54	70	2.39 (1.38–4.14)^∗∗∗^	1.79 (0.65–4.90)

*Religion:*				
Protestant	160	71	1	1
Orthodox	68	21	2.25 (1.70–2.98)^∗∗∗^	0.77(0.12–5.15)
Muslim	6	11	3.24 (1.98–5.28)^∗∗∗^	0.87(0.12–6.34)
Catholic	5	0	0.55 (0.20–1.48)^∗^	0.19 (0.02–1.64)

*Parity:*				
Prmi-para	99	46	1	1
multipara	140	57	2.46 (1.80–3.34)^∗∗∗^	5.68 (1.57–20.73)^∗^

*The number of child alive:*				
1-2	105	49	1	1
3-4	101	37	2.14 (1.58–3.01)^∗∗∗^	0.5 (0.13–2.00)
>4	32	9	2.73 (1.78–3.98)^∗∗∗^	0.92 (0.31–2.73)

*The number of ANC visit:*				
Once	39	24	1	1
Twice	66	34	1.63 (0.97–2.70)	7.93 (0.83–76.17)
Three times	96	26	1.94 (1.28–2.94)^∗∗∗^	7.15 (0.74–69.15)
Four times	36	15	3.69 (3.39–5.69)^∗∗∗^	11.32 (1.14–112.64)^∗^
I have no follow-up	2	4	2.4 (0.93–4.38)	6.90 (0.73–67.02)

*Counseled about breast feeding during ANC follow-up:*				
Yes	169	51	1.58 (1.35–1.85)^∗∗∗^	1.16 (2.59–3.96)^∗^
No	70	52	1	1

*Prior-birth:*				
Yes	231	79	1.78 (1.45–2.17)^∗∗∗^	4.72 (1.69–13.21)^∗∗^
No	8	24	1	1

*History of support from their family about breastfeeding:*				
Yes	174	32	1.43 (1.23–1.66)^∗∗∗^	0.22 (0.11–0.43)^∗∗∗^
No	65	71	1	1

*History of baby illness in the 1^st^ 4 days of delivery:*				
Yes	49	24	1.58 (1.39–1.79)^∗∗∗^	1.77 (0.93–3.39)
No	190	79	1	1

NB: ^∗^ = *p*-value <0.2, ^∗∗^ = *p*-value <0.05, ^∗∗∗^ = *p*-value <0.01

## Data Availability

The data sets used and/or analyzed during the current study are available from the corresponding author on reasonable request.

## Abbreviations

ANC: Anti-natal care
ARI: Acute respiratory infection
CI: Confidence-interval
EBF: Exclusive-breastfeeding
EDHS: Ethiopia-demographic-health-survey
PNC: Post-natal-care
UNICEF: United nation children's fund.
